# Polyphosphate-Functionalized PGMA-Grafted Mesoporous Silica Nanoparticles for Selective U(VI) Removal from Aqueous Solutions

**DOI:** 10.3390/molecules31203664

**Published:** 2026-10-13

**Authors:** Abdulmalik S. Alshammari, Eid M. Alosime, Ahmed A. Basfar

**Affiliations:** 1 MSc in Nuclear Engineering Program, College of Engineering, King Saud University, Riyadh 12372, Saudi Arabia; abdulmalik1211230@gmail.com; 2 King Abdulaziz City for Science and Technology (KACST), P.O. Box 6086, Riyadh 11442, Saudi Arabia; 3 Department of Mechanical Engineering, College of Engineering, King Saud University, Riyadh 12372, Saudi Arabia

**Keywords:** uranium removal, mesoporous-silica nanoparticles, polymer, surface-initiated atom transfer radical polymerization

## Abstract

Uranium(VI) contamination in aquatic environments poses significant environmental and public health concerns, creating a need for efficient and selective adsorbents. In this study, polyphosphate-functionalized poly(glycidyl methacrylate)-grafted mesoporous silica nanoparticles (PP-PGMA/MSNs) were successfully synthesized and evaluated for U(VI) removal from aqueous solutions. A key novelty of this work lies in the incorporation of phosphate-rich functionalities onto PGMA-grafted MSNs prepared via ATRP, together with the systematic investigation of their effects on U(VI) adsorption equilibrium and selectivity in multi-metal systems. Fourier-transform infrared spectroscopy (FTIR), Brunauer–Emmett–Teller (BET), scanning electron microscopy (SEM) and thermogravimetric analysis (TGA) confirmed the successful incorporation of phosphate-containing functional groups onto the MSN surface. The adsorbent exhibited rapid adsorption, reaching equilibrium within 120 min, with optimum performance observed at pH 4–6. A maximum U(VI) removal efficiency of 73.81% was achieved at an initial concentration of 10 mg L^−1^, while the maximum adsorption capacity reached 114.94 mg g^−1^. Equilibrium data were well described by the Langmuir isotherm model (R^2^ = 0.9697), indicating predominantly monolayer adsorption on relatively homogeneous adsorption sites. Despite a substantial reduction in surface area following functionalization, the material maintained a high adsorption capacity, suggesting that uranium uptake is governed primarily by interactions at phosphate-containing active sites. Furthermore, the adsorbent exhibited preferential affinity toward U(VI) in multi-metal systems containing Cr, Ni, Cu, Zn, Cd, and Pb ions, demonstrating enhanced selectivity toward uranyl species. The adsorption behavior suggests that phosphate functionalities serve as the primary binding sites through surface complexation and coordination interactions. These findings demonstrate the potential of PP-PGMA/MSNs as selective adsorbents for U(VI) removal from complex aqueous systems and environmental remediation applications.

## 1. Introduction

The presence of uranium (U) in groundwater has become a major environmental and public health concern due to its chemical toxicity and radiological hazards [1]. U contamination may originate from natural geological formations as well as anthropogenic activities, including U mining, nuclear fuel processing, and the improper disposal of radioactive waste [2,3]. Because groundwater serves as a primary source of drinking water in many regions, the occurrence of U in water resources has attracted increasing attention worldwide. Long-term exposure to U-contaminated water may result in adverse health effects, particularly kidney damage and increased cancer risks [4]. Consequently, the development of efficient and sustainable technologies for U removal from aqueous environments has become an important research priority.

Several techniques have been explored for U(VI) removal from aqueous systems, including reverse osmosis, nanofiltration, capacitive deionization, and adsorption [5,6]. Among these methods, adsorption has received particular attention owing to its operational simplicity, cost-effectiveness, and high removal efficiency [7].

Recent advances in adsorption technology have focused on the development of novel adsorbent materials with enhanced selectivity and capacity for U capture. Mesoporous silica materials are among the most promising adsorbents owing to their high specific surface area, large pore volume, tunable pore sizes, and excellent physicochemical stability [8,9]. These characteristics facilitate the diffusion of U species and improve access to active adsorption sites. Furthermore, the abundant silanol groups present on silica surfaces provide versatile anchoring sites for functional ligands, allowing the design of highly selective adsorbent systems [10].

To improve U adsorption performance, various functional groups have been introduced onto mesoporous silica surfaces. Phosphonate-functionalized mesoporous silica has demonstrated remarkable adsorption capacities and rapid adsorption kinetics for U(VI) removal, particularly under acidic conditions [11]. Similarly, mesoporous silica modified with amidophosphonate ligands exhibited enhanced U extraction behavior as a consequence of strong coordination interactions between phosphorus-containing groups and uranyl ions [12]. The strong affinity of organophosphorus ligands toward actinides has also been reported in conjugated microporous polymers designed for U recovery applications [13]. In addition, adjacent phosphine oxide ligands confined within mesoporous frameworks have shown excellent U-capture performance in acidic media [14]. The effectiveness of phosphorus-containing functionalities has been further confirmed through the successful recovery of U using bisphosphonate-modified mesoporous silica materials [15].

Beyond phosphorus-based functionalization, significant efforts have been devoted to tailoring silica surface properties through the incorporation of other functional groups and hybrid structures. Hybrid mesostructured silica nanoparticles have been developed to improve structural stability and adsorption performance [16], while pyridine-, thiol-, and amine-functionalized mesoporous silicas have demonstrated excellent adsorption behavior toward a range of aqueous pollutants [17]. Surface modification using silane coupling agents represents one of the most versatile approaches for introducing reactive functionalities onto silica materials. In particular, 3-glycidoxypropyltrimethoxysilane (GPTMS) has been widely employed to create epoxy-functionalized silica surfaces suitable for subsequent chemical transformations [18]. Such functionalized mesoporous silica platforms have attracted considerable interest in adsorption and separation applications because of their tunable surface chemistry and structural properties [19].

More recently, surface-initiated atom transfer radical polymerization (ATRP) has emerged as a powerful strategy for engineering advanced mesoporous silica adsorbents. ATRP enables the controlled grafting of polymer brushes onto mesoporous silica nanoparticles, resulting in a high density of accessible functional groups and enhanced adsorption performance [20]. Among the various polymers employed for this purpose, poly(glycidyl methacrylate) (PGMA) is particularly attractive because its pendant epoxy groups can undergo a wide range of post-polymerization modification reactions [21]. This versatility provides an effective route for introducing strongly coordinating ligands onto silica surfaces and creating highly functionalized adsorbent materials.

In parallel with the development of mesoporous silica-based adsorbents, numerous alternative materials have been investigated for U removal from aqueous solutions. Graphene-based materials have demonstrated notable adsorption performance due to their high surface area and abundant functional groups [22,23]. Chitosan-based adsorbents and their magnetic composites have attracted considerable attention because of their biodegradability, low cost, and metal-binding capabilities [24,25,26]. In addition, magnetically separable adsorbents offer practical advantages, including facile recovery and reusability after adsorption processes [27]. Biochar-derived materials have also been explored extensively, and several studies have elucidated the mechanisms responsible for U(VI) uptake by biochar and biochar-based composites [28,29]. Likewise, covalent organic frameworks (COFs) have emerged as highly promising adsorbents because of their ordered porous structures and tunable functionalities [30,31]. Significant progress has additionally been achieved using activated carbon materials [32,33] and functionalized ion-exchange fibers designed for U extraction from mining effluents and contaminated waters [34].

Despite these advances, important challenges remain in developing adsorbents capable of achieving high U selectivity and adsorption efficiency under realistic environmental conditions. In particular, U speciation in aqueous media and the presence of competing ions can significantly influence adsorption mechanisms and overall removal performance. Previous investigations have highlighted the complexity of U sorption behavior on silica-based materials and the critical role of U speciation in governing adsorption processes [35]. In addition to uranium speciation, competition from coexisting ions can significantly affect adsorption mechanisms and overall removal performance. In the present work, phosphate-rich functionalities were introduced onto PGMA-grafted MSNs to provide specific binding sites for uranyl ions, while adsorption equilibrium and selectivity were systematically evaluated in multi-metal systems. Nevertheless, studies focusing on polyphosphate-functionalized mesoporous silica nanoparticles prepared through controlled polymerization strategies remain scarce, and the influence of phosphate-rich functionalities on U(VI) selectivity in multi-metal systems has not been systematically investigated.

Unlike our previous study on ATRP-engineered mesoporous silica nanoparticles (MSNs) for U adsorption [36], this work explores the synthesis of polyphosphate-functionalized PGMA-grafted MSNs (PP-PGMA/MSNs) with phosphate-containing functionalities serving as potential binding sites for U(VI). The study aims to determine how these functionalities influence adsorption capacity, equilibrium behavior and selectivity toward uranium in complex aqueous media. Accordingly, the effects of important operational parameters, including pH, contact time, temperature, adsorbent dosage and initial uranium concentration were evaluated, while adsorption equilibrium was interpreted using Langmuir and Freundlich isotherm models. The selectivity of the developed adsorbent was also investigated in the presence of competing metal ions.

## 2. Results and Discussion

### 2.1. Characterization of Functionalized MSNs

**Figure 1:**
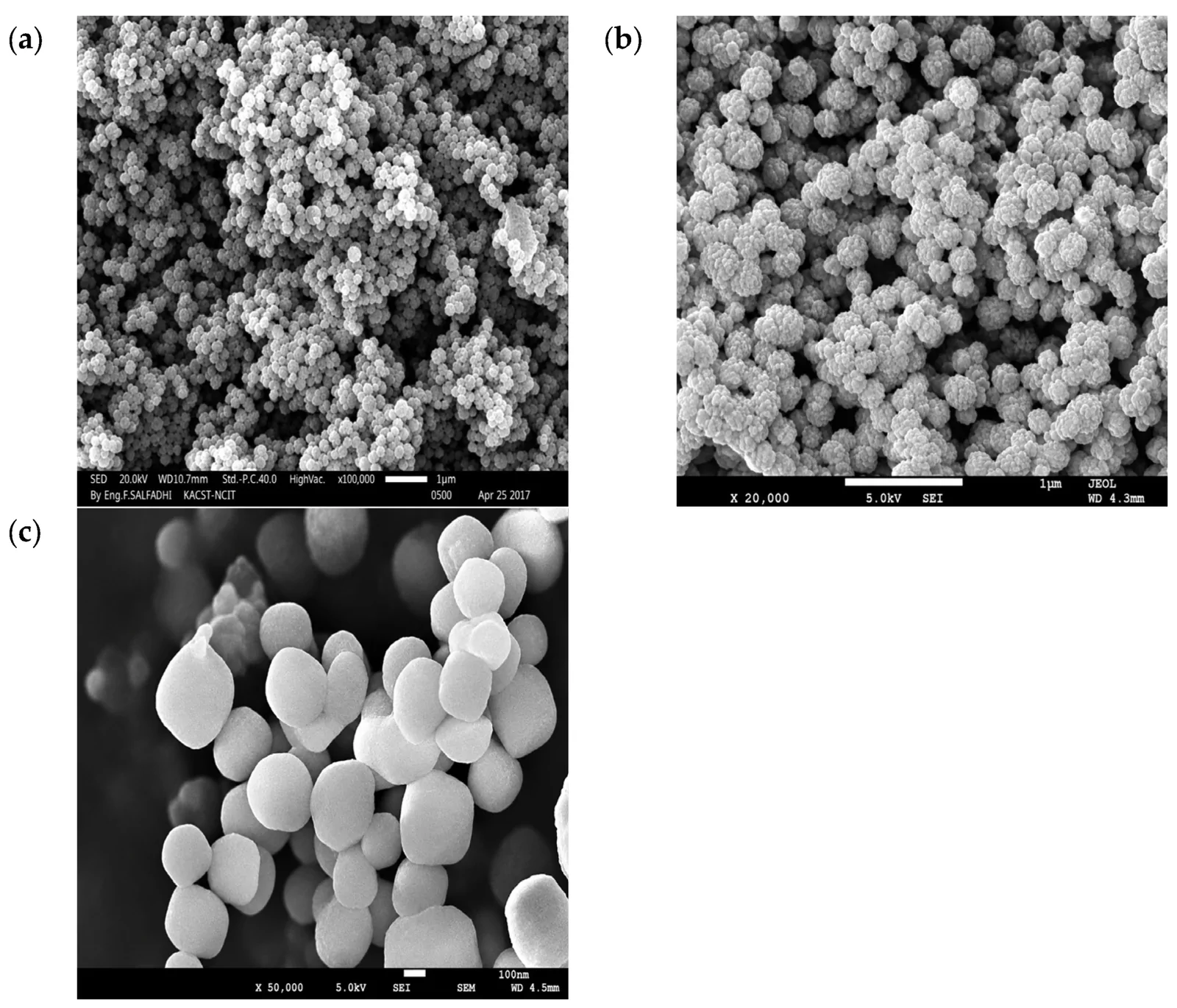
SEM images of prepared mesoporous silica nanoparticles: (**a**) unmodified MSNs (magnification 1 µm ×10,000); (**b**) unmodified MSNs (magnification 1 µm ×20,000) and (**c**) PP-PGMA/MSNs (magnification 100 nm).

**Figure 2:**
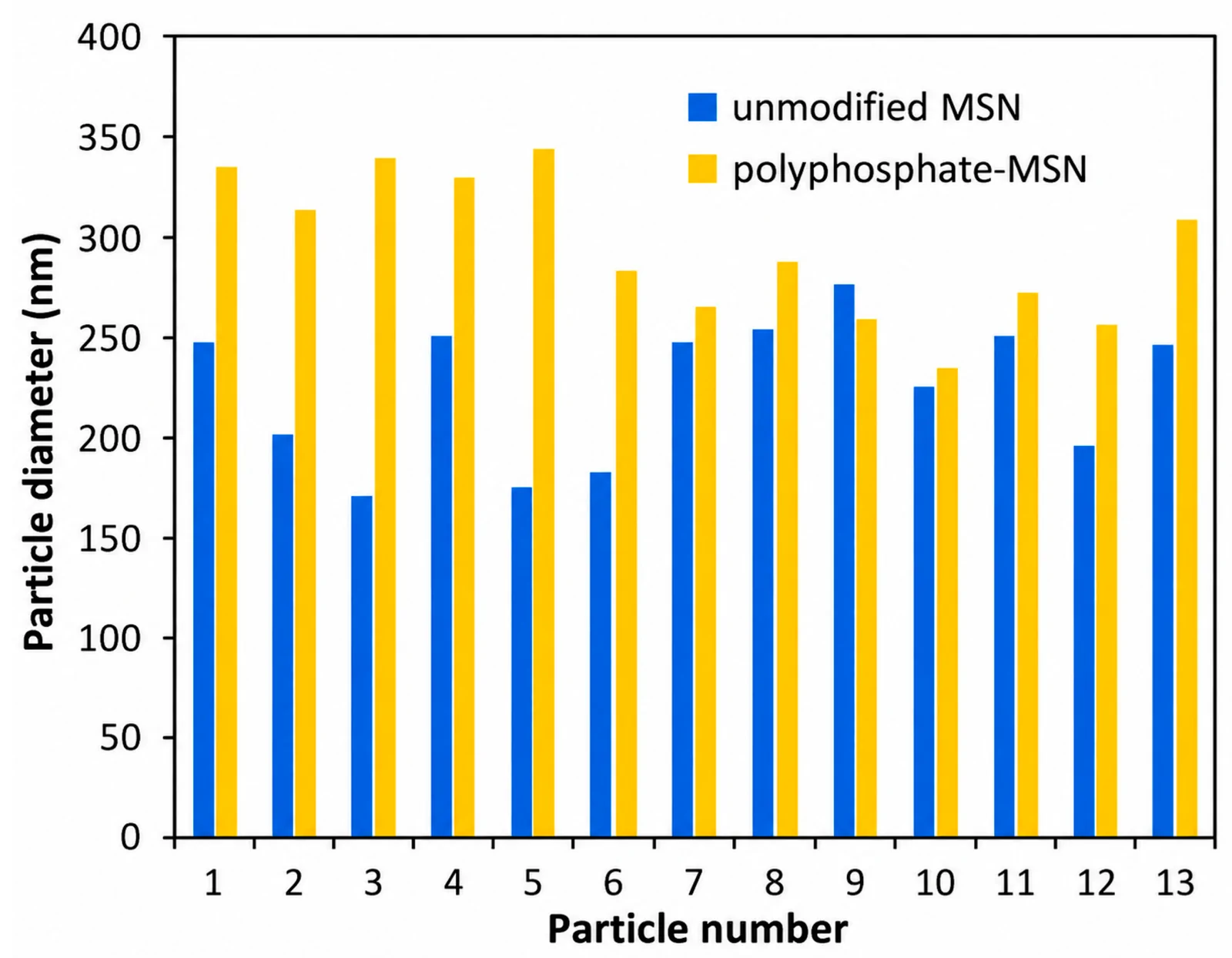
Particle diameter measurements of unmodified and polyphosphate-functionalized MSNs obtained from SEM images using ImageJ software.

The morphology and structural characteristics of the MSNs were examined using scanning electron microscopy (SEM). The MSNs exhibited predominantly spherical morphology, as observed in the SEM image (Figure 1). Analysis of particle diameters using ImageJ software (version 1.54g) (Figure 2) revealed sizes ranging from 150 to 340 nm, with an average diameter of approximately 225 nm for unmodified MSNs (i.e., CTAB-free MSNs prior to surface functionalization). The predominantly spherical morphology indicates the successful formation of MSNs, although slight particle agglomeration was observed. After surface functionalization, the average particle diameter increased to approximately 294 nm. This increase is attributed to the formation of a surface organic layer resulting from the grafting of PGMA chains and phosphate-containing functional groups, confirming successful surface functionalization.

The textural properties of the materials were evaluated by Brunauer–Emmett–Teller (BET) analysis. As summarized in Table 1, the unmodified MSNs exhibited a high specific surface area of 1048 m^2^/g and a pore volume of 1.51 cm^3^ g^−1^, confirming the formation of a well-developed mesoporous structure. Following surface modification, the BET surface area decreased markedly to 10.02 m^2^ g^−1^, while the pore volume was reduced to 0.0166 cm^3^ g^−1^. This substantial decrease is attributed to the grafting of PGMA chains and subsequent incorporation of phosphate-containing functional groups, which occupied and partially blocked the mesoporous channels, thereby limiting nitrogen accessibility during BET measurements. The observed reduction in textural properties is consistent with previous studies on phosphonate-functionalized mesoporous silica materials, where the introduction of organic functionalities resulted in partial pore filling and blockage [37,38]. Interestingly, despite the dramatic reduction in BET surface area from 1048 to 10.02 m^2^ g^−1^ and the corresponding decrease in pore volume, the functionalized material maintained a high U(VI) adsorption capacity of 114.94 mg g^−1^. This behavior indicates that adsorption was not governed primarily by the accessible surface area, as would be expected for a predominantly physical adsorption process. Instead, the results suggest that the incorporation of phosphate-containing functionalities generated highly active binding sites capable of interacting strongly with uranyl ions. Consequently, the enhanced density of chemically active phosphate groups appears to compensate for the loss of accessible porosity, resulting in efficient U(VI) uptake and preferential selectivity in multi-metal systems, consistent with previous reports highlighting the strong affinity of phosphate-based ligands toward U(VI) species [39].

**Figure 3:**
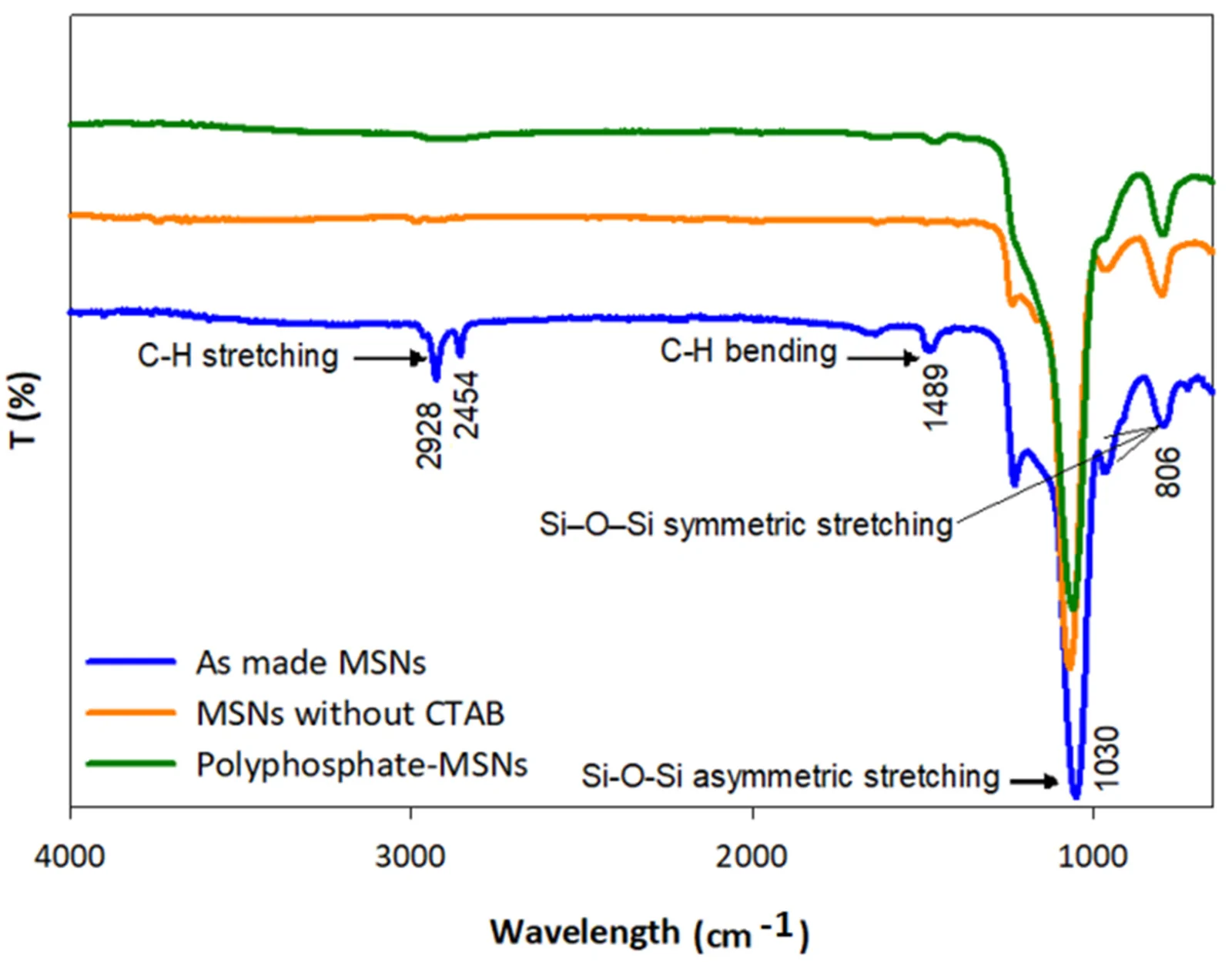
FTIR spectra of as-made MSNs, CTAB-free MSNs and polyphosphate-functionalized MSNs.

Fourier-transform infrared spectroscopy (FTIR) analysis was performed to verify the structural integrity of the mesoporous silica framework and the successful surface functionalization of the MSNs (Figure 3). The FTIR spectrum of the as-made MSNs exhibited characteristic absorption bands associated with both the silica framework and the CTAB template. The broad band in the range of 1030–1240 cm^−1^ was assigned to the asymmetric stretching vibrations of Si-O-Si, while the band at approximately 806 cm^−1^ corresponded to symmetric Si-O-Si stretching. The as-made MSNs also displayed bands at around 2928 and 1489 cm^−1^, attributed to the C-H stretching and bending vibrations of CTAB. After template extraction, these bands disappeared, confirming the effective removal of CTAB while preserving the mesoporous silica structure [20]. Following surface functionalization, changes were observed in the spectral profile of the 1200–900 cm^−1^ region, consistent with the incorporation of phosphate-containing functionalities. However, the characteristic phosphate vibrations overlapped with the intense Si-O-Si absorption bands, making their individual assignment difficult. Similar observations have been reported for phosphonate-functionalized mesoporous silica materials, where phosphate-related vibrations were partially masked by the strong silica absorption bands [38]. Nevertheless, the overall spectral changes, together with the SEM and BET results, provide strong evidence for the successful functionalization of the MSNs.

**Figure 4:**
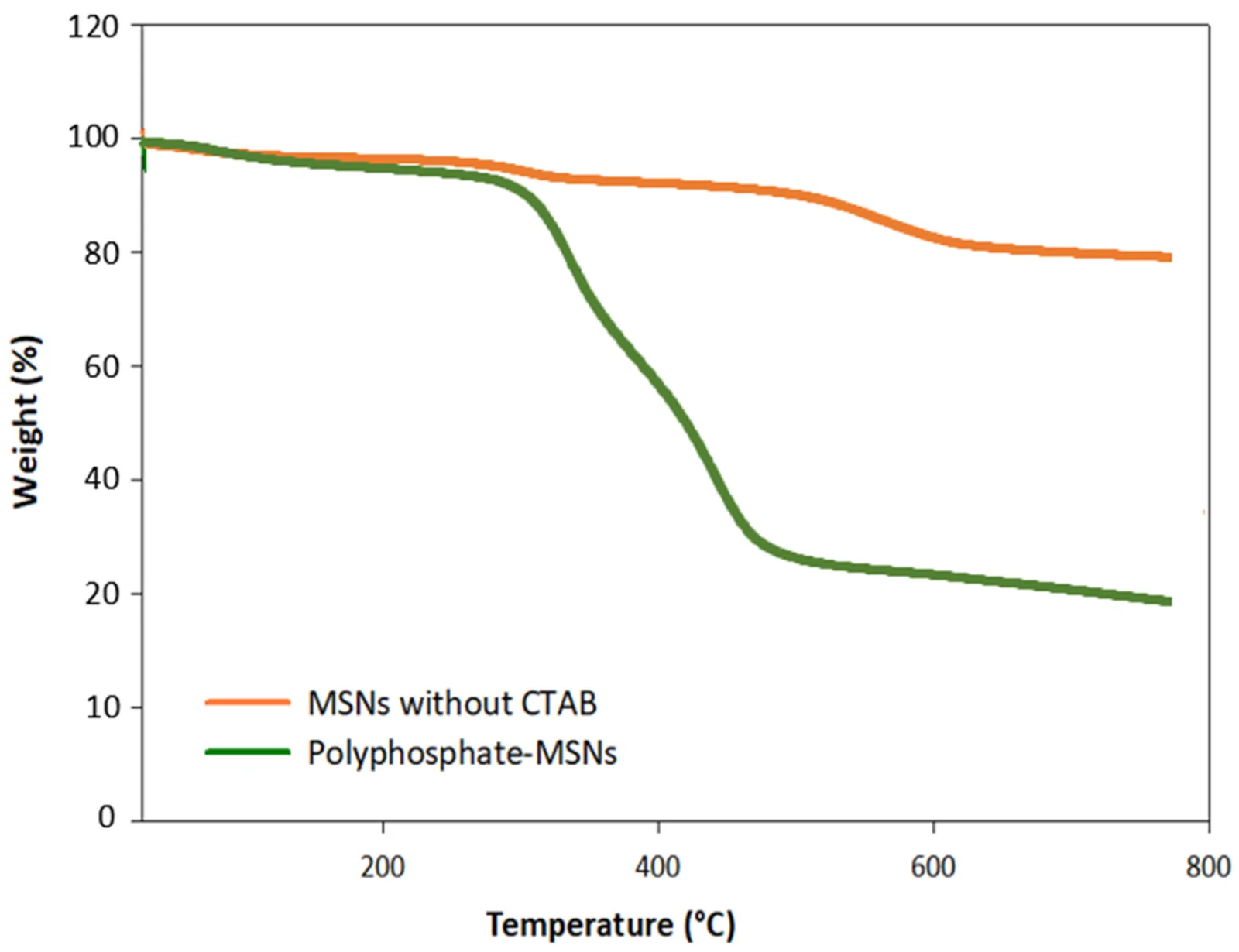
TGA thermograms of unmodified and polyphosphate-functionalized MSNs.

Thermogravimetric analysis (TGA) was performed to evaluate the thermal stability and organic content of the prepared materials (Figure 4). The unmodified MSNs exhibited a relatively small weight loss over the investigated temperature range. In contrast, the polyphosphate-functionalized MSNs displayed a substantially higher weight loss, reaching approximately 24 wt.% upon heating to 800 °C under a nitrogen atmosphere. The major weight-loss region occurred between approximately 250 and 500 °C and is attributed to the decomposition of grafted organic species introduced during the surface functionalization process. The significantly greater weight loss of the functionalized sample compared with the unmodified MSNs confirms the successful incorporation of organic moieties onto the MSN surface, consistent with previous studies on functionalized mesoporous silica materials [37,38].

### 2.2. Adsorption Performance of Polyphosphate-Functionalized MSNs

#### 2.2.1. Effect of Contact Time

The effect of contact time on U adsorption by the polyphosphate-functionalized MSNs was investigated over a period of 5–240 min, and the results are summarized in Table 2A. The adsorption efficiency increased with increasing contact time, indicating the progressive occupation of available adsorption sites on the adsorbent surface. The removal efficiency increased from 24.85% after 5 min to a maximum value of 49.12% after 120 min. This behavior can be attributed to the gradual interaction of U species with active phosphate-containing binding sites on the adsorbent surface. Adsorption equilibrium was reached after approximately 120 min; therefore, this contact time was selected for subsequent experiments.

#### 2.2.2. Effect of Temperature and Initial Concentration

**Figure 5:**
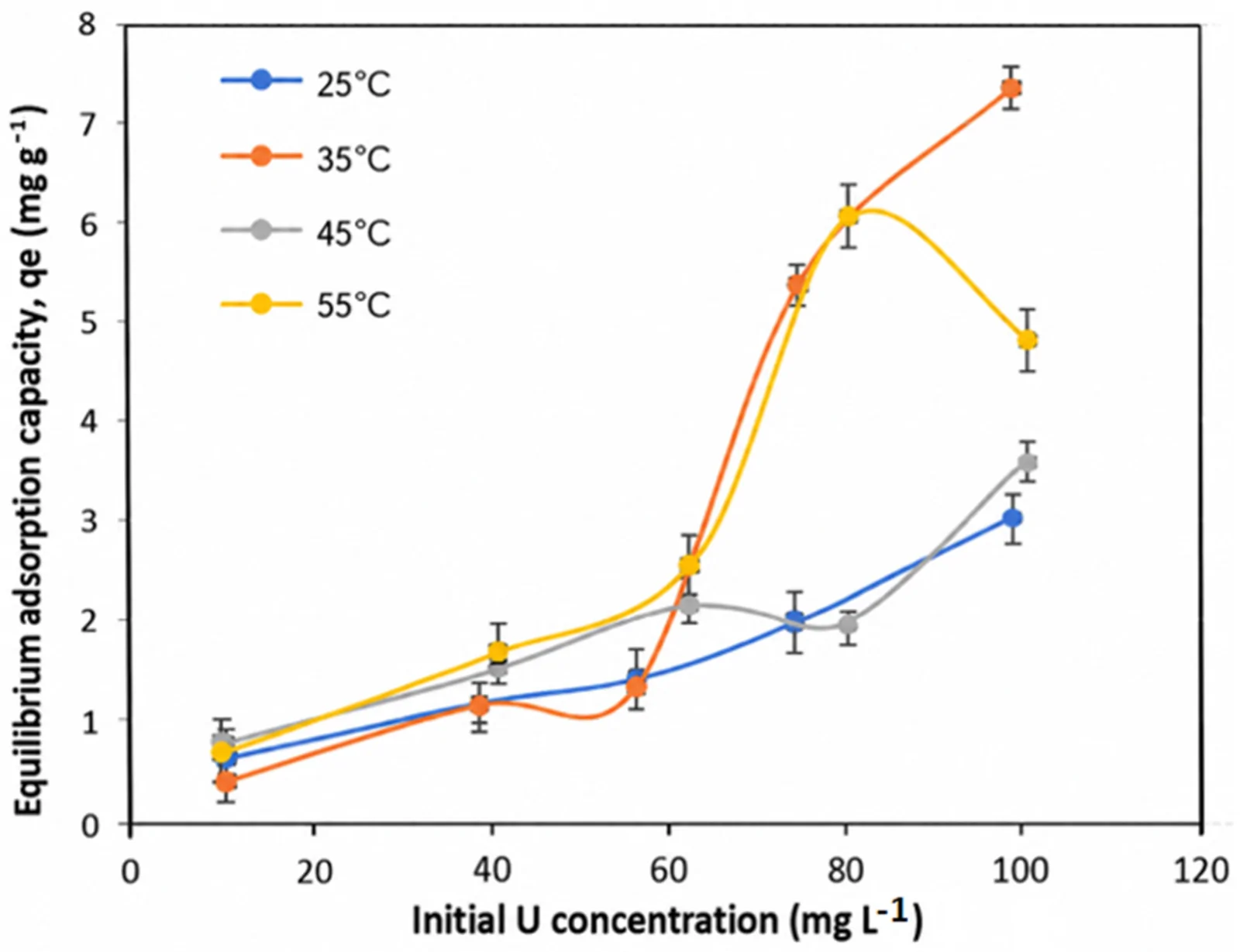
Equilibrium adsorption capacity (qe) as a function of initial U concentration at different temperatures.

**Figure 6:**
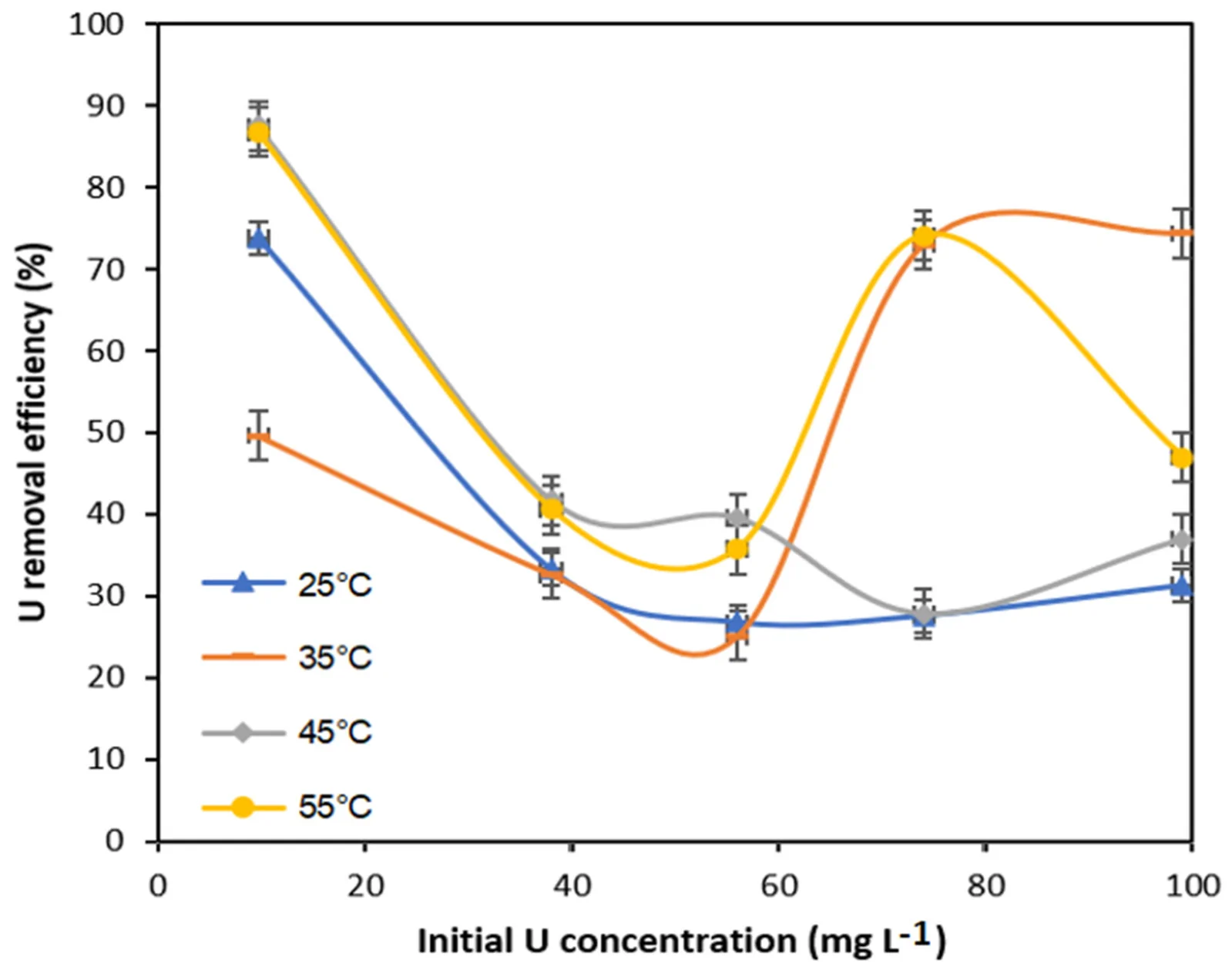
U removal efficiency as a function of initial U concentration at different temperatures.

The effects of temperature and initial U concentration on the adsorption performance of the polyphosphate-functionalized MSNs are summarized in Table 2B–E, while the corresponding adsorption capacities and removal efficiencies are presented in Figure 5 and Figure 6, respectively. At 25 °C, the highest removal efficiency (73.81%) was achieved at an initial U concentration of 10 mg L^−1^, and the efficiency generally decreased with increasing U concentration. Similar behavior was observed at 45 and 55 °C, where the highest removal efficiencies reached 87.53% and 86.70%, respectively, at 10 mg L^−1^ U.

The adsorption behavior varied with temperature and U concentration. In general, increased temperature improved adsorption performance under several experimental conditions, which may be attributed to enhanced diffusion of U species and improved accessibility of active phosphate-containing binding sites. As shown in Figure 5, the adsorption capacity generally increased with increasing U concentration owing to the greater mass transfer driving force. The removal efficiency trends shown in Figure 6 indicate that adsorption performance was influenced by the balance between available adsorption sites and dissolved U species. The highest removal efficiency observed in this study was 87.53% at 45 °C and an initial U concentration of 10 mg L^−1^.

#### 2.2.3. Effect of pH and U Speciation

**Figure 7:**
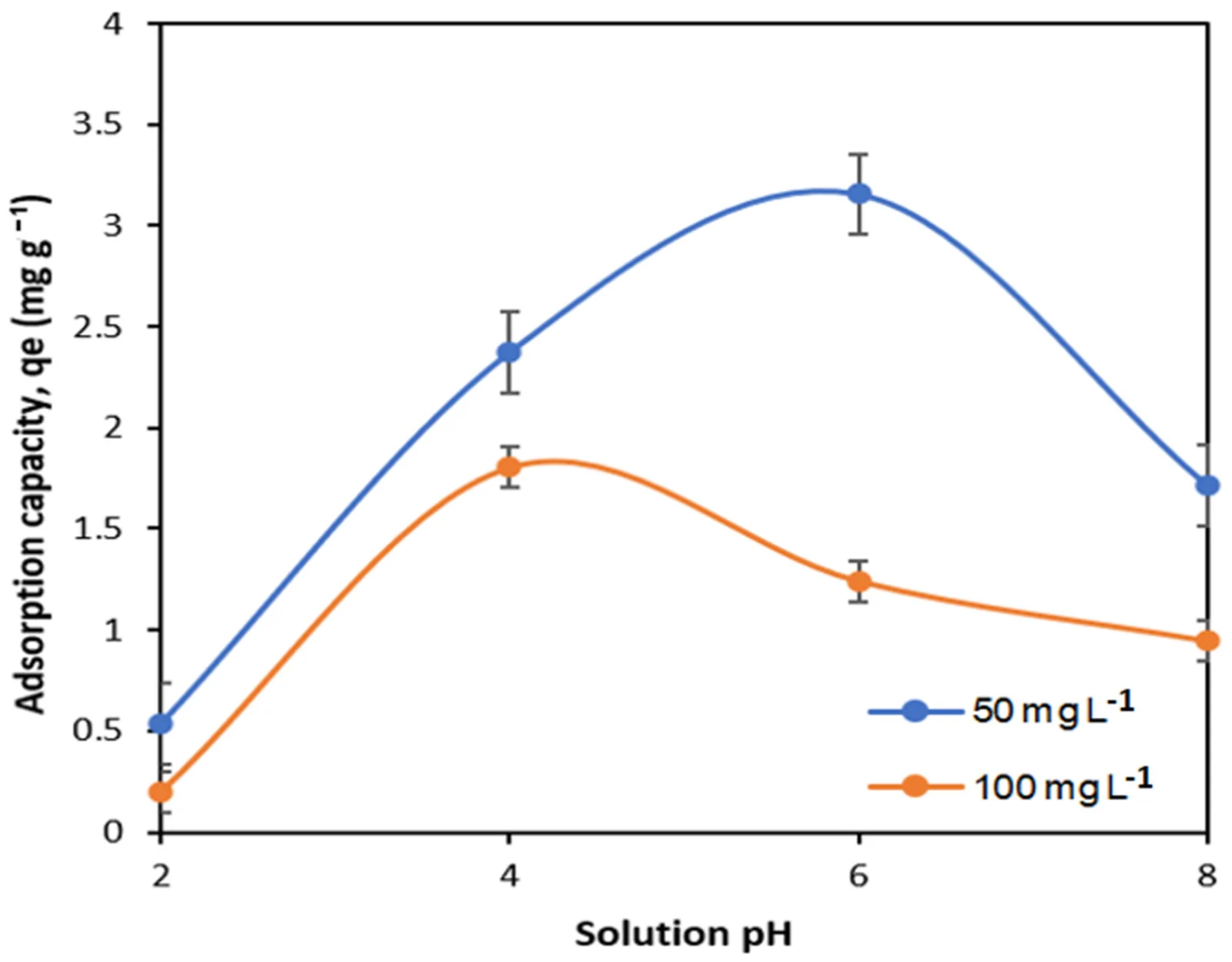
Equilibrium adsorption capacity (qe) as a function of solution pH.

**Figure 8:**
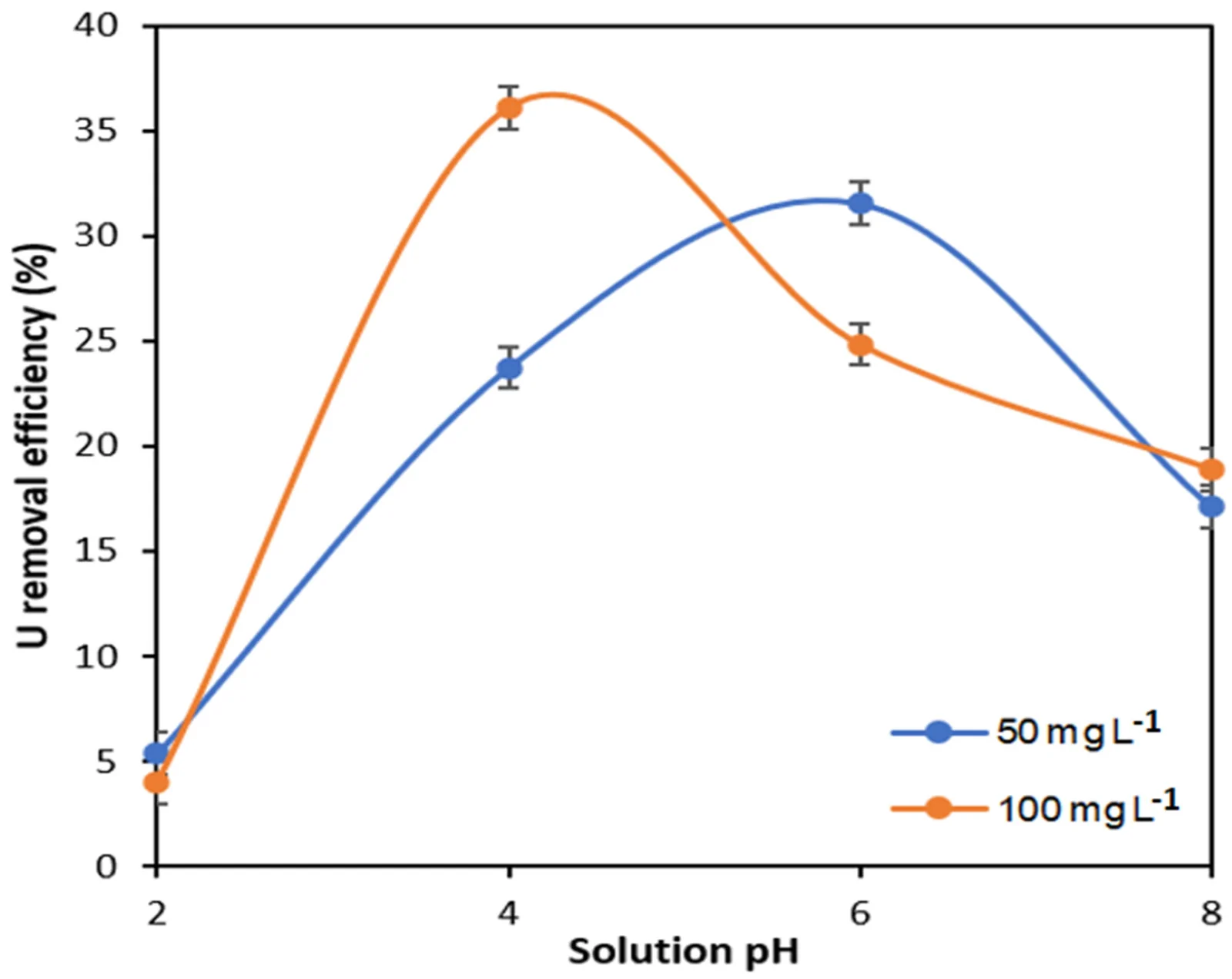
U removal efficiency (%) as a function of solution pH.

The effect of solution pH on U adsorption by the polyphosphate-functionalized MSNs is summarized in Table 3, while the corresponding adsorption capacities and removal efficiencies are presented in Figure 7 and Figure 8, respectively. Solution pH significantly influenced the adsorption performance of the developed adsorbent. At pH 2, very low removal efficiencies were observed for both initial U concentrations, reaching only 5.36% and 4.00% for 100 and 50 mg L^−1^, respectively. This behavior can be attributed to competition between H^+^ ions and dissolved U species for the available phosphate-containing binding sites. As the pH increased from 2 to 4–6, the adsorption performance improved considerably. The maximum removal efficiencies were obtained at pH 6 for 100 mg L^−1^ U (31.55%) and at pH 4 for 50 mg L^−1^ U (36.10%). This enhancement may be attributed to reduced competition from H^+^ ions and more favorable interactions between dissolved U species and the phosphate-containing functional groups on the adsorbent surface. Further increases in pH to 8 resulted in lower adsorption efficiencies for both U concentrations. This decrease may be associated with changes in U(VI) speciation and adsorption equilibrium at alkaline conditions, indicating that pH 4–6 provides the most favorable conditions for U adsorption [28].

#### 2.2.4. Effect of Adsorbent Dosage

The effect of adsorbent dosage on U adsorption by the polyphosphate-functionalized MSNs was investigated at room temperature for 60 min using initial U concentrations of 50 and 100 mg L^−1^. The results are summarized in Table 4. For the 100 mg L^−1^ U solution, the removal efficiency ranged from 31.55% to 38.44%, with the highest value obtained at an adsorbent dosage of 30 mg. Similarly, for the 50 mg L^−1^ U solution, the highest removal efficiency (39.74%) was also achieved at 30 mg. The improved adsorption performance at this dosage may be attributed to the increased availability of active phosphate-containing binding sites. However, further increasing the adsorbent dosage to 50 mg did not improve the removal efficiency, suggesting that the additional adsorption sites were not effectively utilized under the investigated conditions. Therefore, an adsorbent dosage of 30 mg was selected as the optimum dosage under the investigated conditions.

### 2.3. Selectivity in Multi-Metal Systems

The selectivity of the polyphosphate-functionalized MSNs toward U was evaluated in the presence of competing metal ions, including Cr, Ni, Cu, Zn, Cd, and Pb (Table 5A). In the multi-metal system, the adsorbent exhibited the highest removal efficiency for U (40%), whereas the removal efficiencies of the competing metal ions ranged from 9% to 18%. These results demonstrate a preferential affinity of the adsorbent toward U in comparison with the investigated competing ions. To further assess the effect of metal-ion competition, an additional experiment was conducted in the absence of Pb (Table 5B). Under these conditions, the removal efficiency of U remained relatively high (37%), indicating that the selectivity of the adsorbent toward U was largely preserved even when the composition of the competing-ion system was modified. The slight decrease in U removal efficiency suggests that the adsorption behavior is primarily governed by the interaction between U species and the phosphate-containing functional groups on the adsorbent surface. The observed selectivity can be attributed to the strong affinity of U(VI) species toward oxygen-donor ligands, particularly phosphate groups, which promote the formation of stable surface complexes. Consequently, the polyphosphate-functionalized MSNs exhibited preferential selectivity for U in mixed-metal systems, highlighting their potential applicability for uranium removal from complex aqueous media.

### 2.4. Adsorption Isotherm Analysis: Langmuir and Freundlich Models

Adsorption isotherms describe the equilibrium relationship between the adsorbate and the adsorbent, providing valuable information regarding adsorption mechanisms, surface properties, and the nature of adsorbate–adsorbent interactions. In the present study, equilibrium adsorption experiments were conducted at room temperature to eliminate thermal effects and ensure that the observed variations in adsorption behavior were solely associated with changes in the equilibrium concentration of U(VI). The equilibrium data were analyzed using the linear forms of the Langmuir and Freundlich isotherm models to evaluate the adsorption performance of the polyphosphate-functionalized MSNs.

#### 2.4.1. Langmuir Model

The Langmuir adsorption isotherm assumes monolayer adsorption on a homogeneous surface containing a finite number of energetically equivalent adsorption sites, with no interaction between adjacent adsorbed molecules [40]. The linearized Langmuir equation is expressed as:

$$\frac{C_{e}}{q_{e}}=\frac{1}{q_{max}K_{L}}+\frac{C_{e}}{q_{max}}$$

where $C_{e}$ (mg L^−1^) is the equilibrium concentration of U(VI), $q_{e}$ (mg g^−1^) is the equilibrium adsorption capacity, $q_{max}$ (mg g^−1^) is the maximum monolayer adsorption capacity, and $K_{L}$ (L mg^−1^) is the Langmuir adsorption constant related to the affinity of the adsorbent toward the adsorbate.

**Figure 9:**
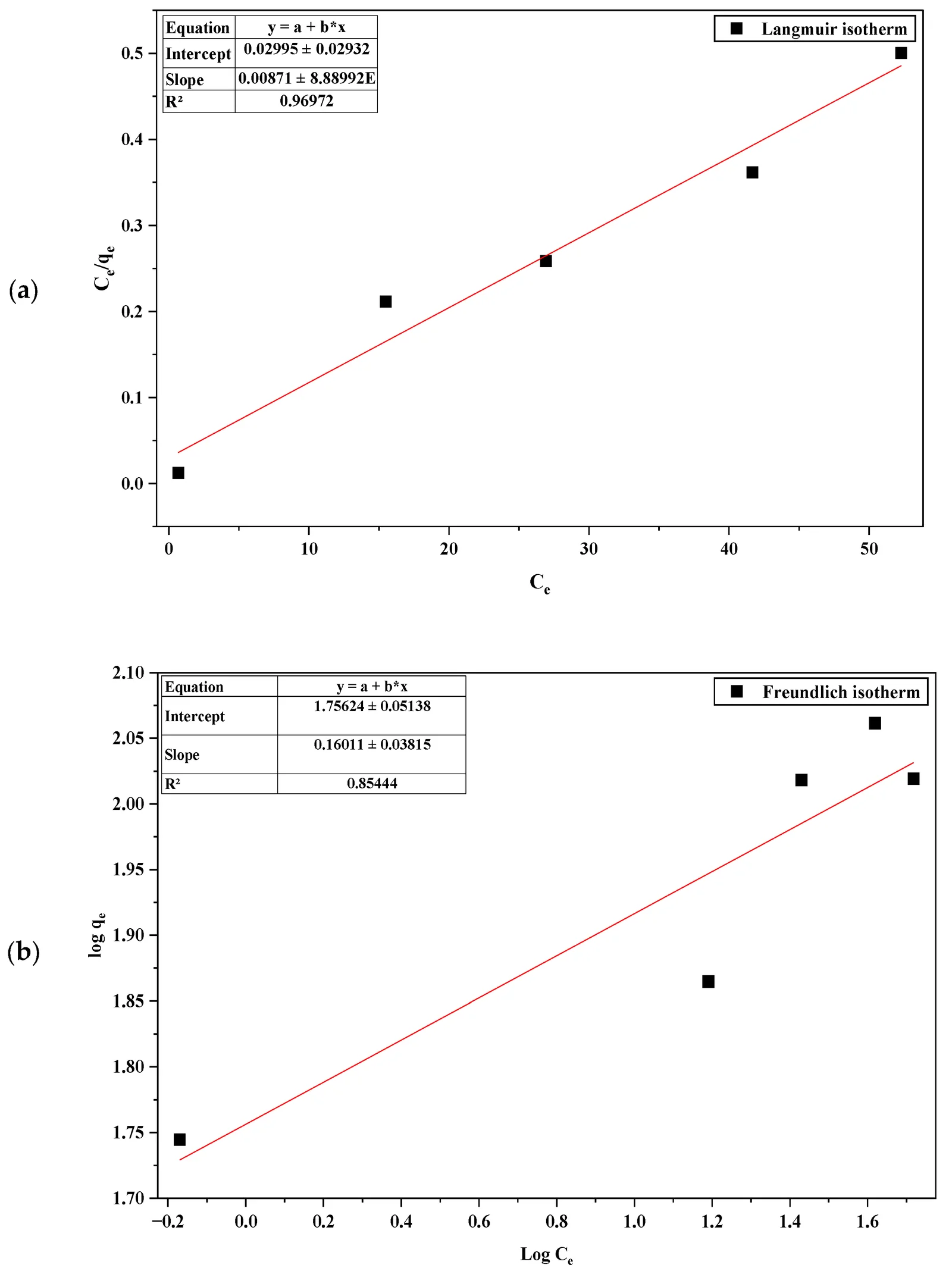
Linearized Langmuir (**a**) and Freundlich (**b**) adsorption isotherms for U(VI) adsorption onto polyphosphate-functionalized MSNs.

The Langmuir parameters were obtained from the linear plot of $C_{e}/q_{e}$ versus $C_{e}$ (Figure 9a). The calculated $q_{max}$ was 114.94 mg g^−1^, while the Langmuir constant was 0.29 L mg^−1^, indicating a strong affinity between U(VI) ions and the polyphosphate-functionalized MSN surface. Furthermore, the excellent correlation coefficient (R^2^ = 0.9697) demonstrates that the Langmuir model adequately describes the equilibrium adsorption behavior, suggesting that uranium adsorption predominantly occurs through monolayer coverage on relatively homogeneous adsorption sites.

The superior agreement with the Langmuir model can be attributed to the successful surface functionalization of the MSNs with phosphate-containing groups. As demonstrated by the FTIR analysis, phosphate functionalities were successfully introduced onto the silica surface, providing chemically similar coordination sites for uranyl ions. Moreover, SEM analysis revealed an increase in particle diameter after functionalization, confirming the formation of a surface organic layer, while BET measurements showed a substantial reduction in specific surface area and pore volume due to pore occupation by PGMA chains and phosphate groups. Although the accessible surface area decreased considerably after functionalization, the adsorption capacity remained remarkably high. This finding indicates that U(VI) adsorption is governed primarily by the chemical affinity of the phosphate functional groups rather than by the available physical surface area. The phosphate oxygen atoms are believed to act as the principal adsorption sites for uranyl ions through coordination and surface complexation interactions, thereby contributing significantly to the observed adsorption capacity.

The favorability of adsorption was further evaluated using the dimensionless separation factor ($R_{L}$):

$$R_{L}=\frac{1}{1+K_{L}C_{0}}$$

where $C_{0}$ is the initial U(VI) concentration (mg L^−1^). The calculated $R_{L}$ values ranged from 0.04 to 0.13 (Table 6), confirming highly favorable adsorption since $0<R_{L}<1$ indicates favorable adsorption behavior [40]. The relatively low $R_{L}$ values further demonstrate the strong interaction between uranium ions and the phosphate-functionalized adsorption sites.

#### 2.4.2. Freundlich Model

The Freundlich isotherm is an empirical model commonly employed to describe adsorption on heterogeneous surfaces with non-uniform adsorption energies. The linearized Freundlich equation is given by

$$\;\log q_{e}=\log K_{F}+\frac{1}{n}\log C_{e}$$

where $K_{F}$ is the Freundlich adsorption constant and n represents the adsorption intensity and surface heterogeneity.

The Freundlich parameters were obtained from the linear plot of $\log q_{e}$ versus $\log C_{e}$ (Figure 9b). The calculated constants were $K_{F}=57.04$ mg g^−1^ and n=6.246. Since the value of n lies between 1 and 10, the adsorption process can be considered highly favorable. Moreover, the relatively high n value indicates strong adsorption intensity and suggests that the functionalized MSNs possess a high affinity toward U(VI) ions over the investigated concentration range.

However, the Freundlich model produced a lower correlation coefficient (R^2^ = 0.8544) than the Langmuir model, indicating that surface heterogeneity plays a less significant role in describing the adsorption equilibrium. This observation suggests that the introduced phosphate functional groups are relatively uniformly distributed on the MSN surface, resulting in adsorption sites with comparable binding energies.

Comparison of the two isotherm models clearly demonstrates that the Langmuir model provides a significantly better description of the equilibrium data than the Freundlich model. This behavior suggests that U(VI) adsorption onto the polyphosphate-functionalized MSNs predominantly occurs through monolayer adsorption on chemically homogeneous active sites. Such behavior is consistent with the successful grafting of phosphate-containing functional groups confirmed by FTIR analysis, which introduced specific coordination sites for uranyl ions. Although BET analysis revealed a dramatic reduction in surface area and pore volume following surface functionalization, the adsorption capacity remained high ($q_{max}$ = 114.94 mg g^−1^). The introduced phosphate groups are capable of forming stable coordination complexes with U(VI), resulting in strong adsorption affinity as reflected by the relatively high K_L_ value and the favorable RL values. Consequently, the adsorption process is dominated by surface complexation at phosphate active sites instead of nonspecific physical adsorption.

**Figure 10:**
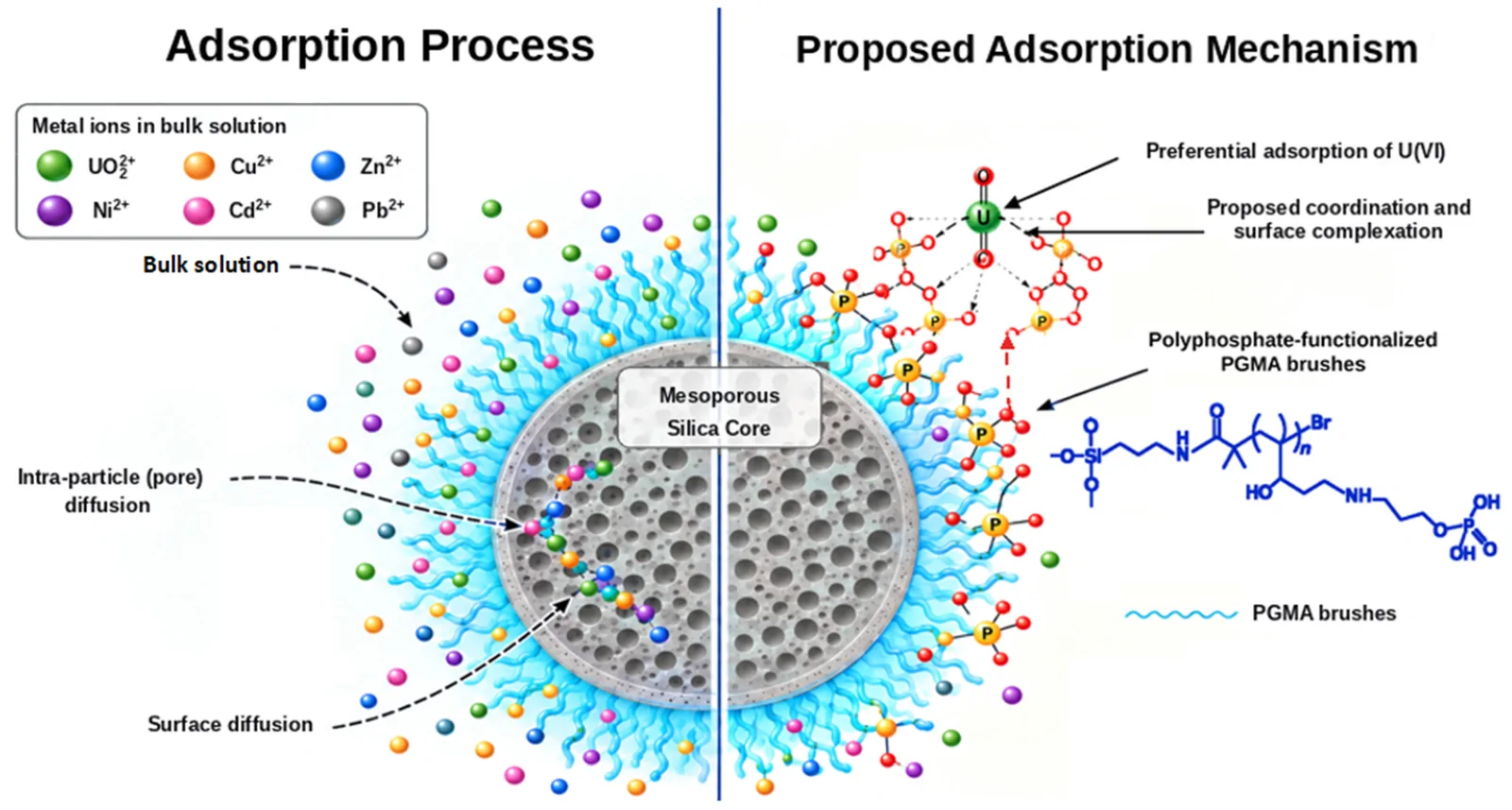
Proposed mechanism for U(VI) adsorption onto PP-PGMA/MSNs.The $q_{max}$ = 114.94 mg g^−1^, together with the favorable RL and n values, confirms the effective adsorption performance of the synthesized PP-PGMA/MSNs toward U(VI). As shown in Table 7, the adsorption capacity of the developed material is comparable to those reported for several phosphate-functionalized adsorbents in the literature [11,38,40,41]. Although some highly functionalized silica-based materials exhibit higher adsorption capacities, the PP-PGMA/MSNs developed in this work offer the additional advantage of phosphate-rich active sites introduced through ATRP-assisted PGMA grafting and subsequent phosphate functionalization, together with preferential U(VI) selectivity in multi-metal systems. These characteristics highlight the novelty of the developed material and demonstrate the effectiveness of phosphate-rich functionalization for selective U(VI) adsorption in complex aqueous environments.

Based on the experimental results, a proposed mechanism for U(VI) adsorption onto PP-PGMA/MSNs is illustrated in Figure 10. U(VI) ions are transported from the bulk solution to the adsorbent surface and mesoporous channels through diffusion processes. Adsorption is proposed to occur primarily through coordination and surface complexation interactions between uranyl ions and phosphate-rich active sites distributed along the PGMA-grafted layer, resulting in preferential U(VI) adsorption in the presence of competing metal ions.

**Table 7:** Comparison of U(VI) adsorption capacities of PP-PGMA/MSNs with selected adsorbents reported in the literature.

Reference	Absorbent	$q_{max}$	pH
This study	PP-PGMA/MSNs	114.94	4–6
[11]	Phosphorus-doped (8.3 wt.%) mesoporous silica	345	4
[38]	SBA-15 functionalized with phosphonate	217	4
[41]	Phosphate functionalized bacterial cellulose	50.65	4–8
[42]	Phosphonate-functionalized mesoporous silica (NP10)	303	1.5–8

## 3. Materials and Methods

### 3.1. Materials

Cetyltrimethylammonium bromide (CTAB, 98%), tetraethyl orthosilicate (TEOS, 98%), 3-aminopropyltriethoxysilane (APTES, >98%), O-phosphorylethanolamine (OPEA, 98%), 3-glycidoxypropyltrimethoxysilane (GPTMS, 98%) and Glycidyl methacrylate (GMA, ≥97.0%, GC), were purchased from Sigma-Aldrich (St. Louis, MO, USA). Copper(I) chloride (CuCl), copper(II) bromide (CuBr_2_), and 2,2′-bipyridine (Bipy) were used as ATRP catalyst components. 2-bromoisobutyryl bromide (BIBB, 98%), Triethylamine (Et_3_N) and dichloromethane (CH_2_Cl_2_) were used for initiator immobilization. Organic solvents including hexane, methanol, ethanol, isopropanol (IPA), and toluene were of analytical/HPLC grade. Dimethyl sulfoxide (DMSO) and hydrochloric acid (HCl) were obtained from Fisher Scientific (Waltham, MA, USA). A stock solution of uranium (UO_2_^2+^, 900 mg L^−1^) was prepared from uranyl nitrate hexahydrate. Competing metal ions (Cr, Ni, Cu, Zn, Cd, Pb) were obtained from Merck (Darmstadt, Germany) as nitrate salts. Deionized water (18.2 MΩ·cm) was obtained from an Elga Pure Nanopore system. All reagents were used as received without further purification.

### 3.2. Synthesis of Polyphosphate-Functionalized PGMA-Grafted MSNs (PP-PGMA/MSNs)

**Figure 11:**
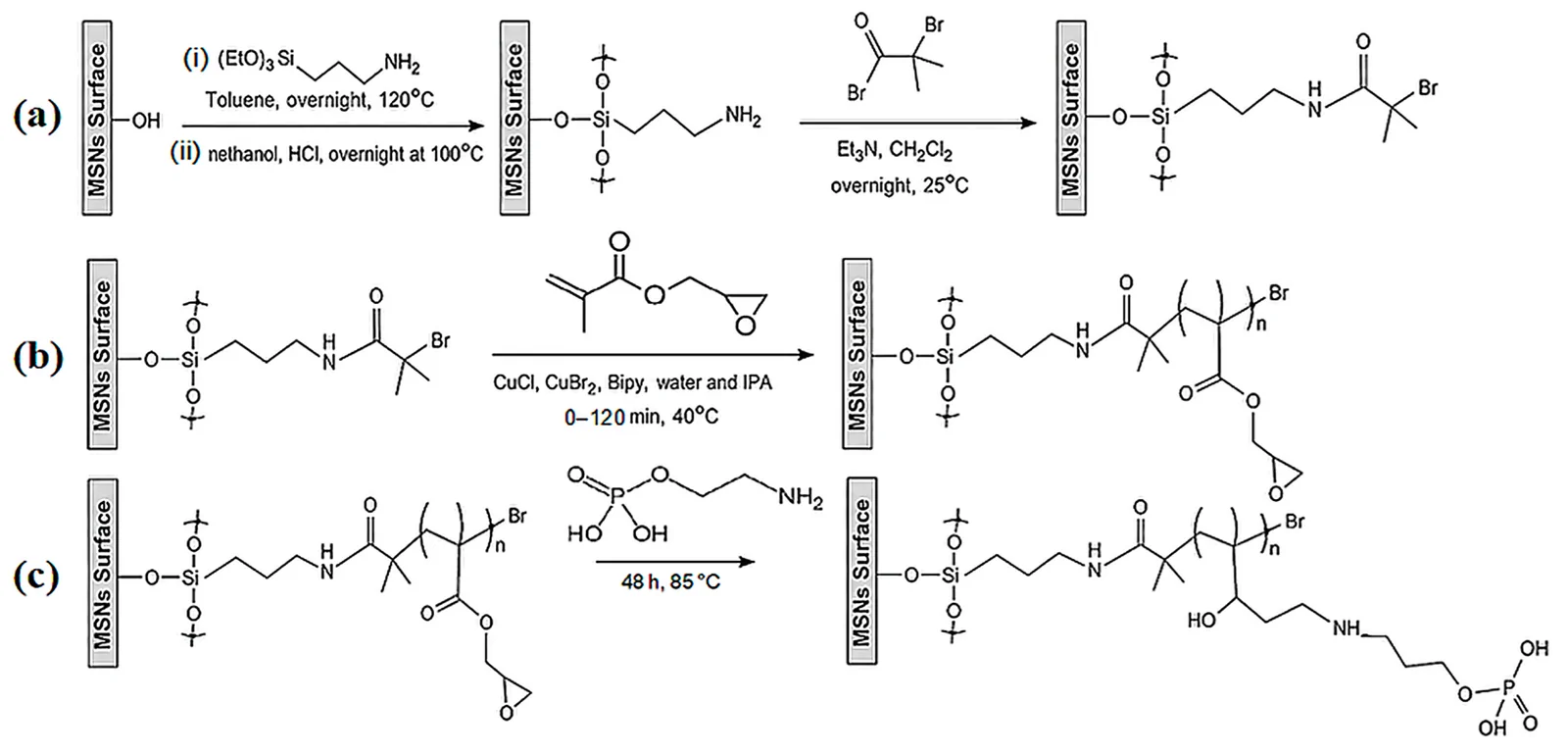
Schematic illustration of the synthesis of polyphosphate-functionalized polymer-grafted MSNs. (**a**) Preparation of ATRP initiator-functionalized MSN–Br. (**b**) Surface-initiated ATRP growth of PGMA brushes from the MSN surface. (**c**) Post-functionalization of PGMA brushes to yield polyphosphate-functionalized MSNs. Adapted from Alswieleh et al. [20] and Alshammari et al. [36].

The synthesis procedure was adapted from the methods reported by Alswieleh et al. [20] and Alshammari et al. [36], with appropriate modifications, as illustrated in Figure 11.

#### 3.2.1. Synthesis of MSNs

MSNs were synthesized via a surfactant-templated sol–gel method using CTAB as a structure-directing agent. Briefly, 1 g of CTAB was dissolved in 160 mL of deionized water. Upon the addition of 7 mL of 28% ammonia solution, a clear homogeneous solution was obtained. Subsequently, a mixture of 20 mL n-hexane and 5 mL TEOS was added dropwise over 30 min under continuous stirring. The reaction mixture was maintained at 35 °C and gradually evolved into a milky colloidal suspension. After 12 h of stirring, the resulting MSNs were collected by centrifugation, washed thoroughly with deionized water and ethanol, and dried at 100 °C for 2 h. The removal of the surfactant template (CTAB) was carried out by dispersing 1.5 g of obtained MSNs in 160 mL of methanol, followed by the addition of 9 mL of concentrated HCl (12 M). The mixture was refluxed for 24 h to ensure complete extraction of the template. This extraction process was repeated, after which the nanoparticles were collected by centrifugation, washed several times with ethanol, and dried for 12 h.

#### 3.2.2. Preparation of 3-Aminopropyl-Functionalized MSNs (AP-MSNs)

AP-MSNs were prepared through surface modification of MSNs using APTES. In a typical synthesis, 1.5 g of obtained MSNs were suspended in 50 mL of dry toluene containing 2.5 mmol of APTES. The reaction mixture was heated under reflux for 24 h to facilitate the grafting of aminopropyl groups onto the silica surface. Following the reaction, the AP-MSNs were separated by centrifugation and washed five times with water and five times with methanol to eliminate excess silane and other impurities. The purified nanoparticles were then dried in an oven to obtain the final product.

#### 3.2.3. Immobilization of ATRP Initiator

The ATRP initiator was immobilized by reacting the amino groups on the MSN surface with BiBB in the presence of Et_3_N in CH_2_Cl_2_. The reaction was conducted at room temperature overnight under continuous stirring, resulting in bromine-terminated MSNs (MSN–Br), which serve as initiation sites for controlled radical polymerization.

#### 3.2.4. Surface-Initiated ATRP

Surface-initiated ATRP was carried out to grow polymer chains from the MSN surface. The initiator-functionalized MSNs were dispersed in a mixed solvent system of water and IPA. GMA was used as the monomer to introduce epoxy-functionalized polymer chains. The polymerization was performed in the presence of CuCl, CuBr_2_, and Bipy as ligand at 40 °C for 120 min. During this step, poly(glycidyl methacrylate) (PGMA) chains bearing epoxy groups were grafted from the MSN surface, forming polymer-grafted MSNs (MSN-g-PGMA).

#### 3.2.5. Post-Functionalization with Phosphate Groups

The polymer-grafted MSNs were further functionalized with phosphate groups. The material was dispersed in 20 mL DMSO and heated to 60 °C for 1 h. Subsequently, OPEA (3 g dissolved in 10 mL water) was added, and the reaction mixture was stirred at 85 °C for 48 h. During this step, the epoxy groups along the PGMA chains underwent nucleophilic ring-opening reactions with OPEA, leading to the covalent incorporation of phosphate functionalities. The final PP-PGMA/MSNs were collected by centrifugation, washed repeatedly with deionized water and methanol, and dried at 50 °C overnight.

### 3.3. Preparation of Uranium Solutions

Working solutions of UO_2_^2+^ at concentrations of 10, 50, and 100 mg L^−1^ were prepared by dilution of a 900 mg L^−1^ stock solution using the relation C_1_V_1_ = C_2_V_2_.

### 3.4. Characterization and Batch Adsorption Experiments

The synthesis and surface functionalization of MSNs were characterized using several analytical techniques. The specific surface area of the nanoparticles was determined using a Gemini 2375 surface area analyzer (Micromeritics, Norcross, GA, USA) after degassing under vacuum at 150 °C. The BET surface area was calculated over a relative pressure (P/P_0_) range of 0.05–0.25, and the total pore volume was obtained from the amount of N_2_ adsorbed at P/P_0_ = 0.99.

FTIR spectra were recorded using a Nicolet™ iS™ 10 FTIR spectrometer (Thermo Fisher Scientific, Waltham, MA, USA) over the range of 4000–400 cm^−1^ to identify the surface functional groups and confirm the modification steps. Thermal stability was evaluated using TGA with an SII TGA 6300 instrument (Seiko Instruments Inc., Torrance, CA, USA) under a nitrogen atmosphere. The morphology and particle size of the synthesized nanoparticles were examined by SEM (Jeol JSM-6380, Tokyo, Japan). U concentrations before and after adsorption were determined using inductively coupled plasma mass spectrometry (ICP-MS). Samples for ICP-MS analysis were acidified with diluted nitric acid prior to measurement.

Batch adsorption experiments were conducted to evaluate the effects of contact time, temperature, pH, adsorbent dosage, and competing ions on U removal. For contact time studies, 20 mg of adsorbent was mixed with 50 mL of U solution (100 mg L^−1^, pH 6) and agitated in a thermostatic water bath at 25 °C for different time intervals. The effect of temperature was investigated using 20 mg of adsorbent and U solutions of different initial concentrations over a temperature range of 25–55 °C. The influence of pH was examined using 20 mg of adsorbent and 50 mL of U solution (100 mg L^−1^) over a range of pH values at room temperature. The effect of adsorbent dosage was evaluated using different masses of adsorbent with U solutions of 50 and 100 mg L^−1^. Competitive adsorption experiments were performed using two multi-element solutions containing U and selected competing metal ions. Following adsorption, the suspensions were centrifuged using a Hettich ROTINA 35 centrifuge (Andreas Hettich GmbH & Co. KG, Tuttlingen, Germany), and the supernatants were acidified with 1% HNO_3_ prior to ICP-MS analysis. All experiments were performed under continuous agitation to ensure homogeneous mixing and reproducible adsorption conditions.

## 4. Conclusions

Polyphosphate-functionalized PGMA-grafted MSNs (PP-PGMA/MSNs) were successfully synthesized and applied for the removal of U(VI) from aqueous solutions. The developed adsorbent exhibited rapid adsorption behavior, reaching equilibrium within 120 min, and demonstrated effective U(VI) removal over a wide range of operating conditions. Adsorption performance was strongly influenced by solution pH, temperature, adsorbent dosage, and initial U(VI) concentration, with optimum adsorption observed under mildly acidic conditions. Adsorption equilibrium was best described by the Langmuir isotherm model, indicating predominantly monolayer adsorption on relatively homogeneous adsorption sites. The maximum adsorption capacity of 114.94 mg g^−1^ demonstrates the strong affinity of the synthesized adsorbent toward U(VI). Characterization analyses verified the successful surface functionalization of the MSNs. FTIR analysis confirmed the incorporation of phosphate-containing functional groups, while BET analysis revealed a reduction in surface area and pore volume following functionalization. Despite this decrease in accessible porosity, the adsorption performance remained high, indicating that U(VI) uptake appears to be governed primarily by coordination interactions at phosphate-containing active sites rather than by the accessible surface area alone. The synthesized adsorbent also exhibited preferential selectivity toward U(VI) in mixed-metal systems, demonstrating its potential applicability for uranium removal from complex aqueous environments. The integration of surface-initiated ATRP with post-polymerization phosphate functionalization enabled the generation of phosphate-rich active sites on MSNs and provided insight into their role in U(VI) adsorption equilibrium and selectivity in multi-metal systems. Overall, the results indicate that PP-PGMA/MSNs represent a promising adsorbent for efficient and selective U(VI) remediation and offer considerable potential for future water treatment applications.

## Figures and Tables

**Table 1:** Physiochemical data for polyphosphate-MSNs samples.

Material	BET Surface Area (m^2^ g^−1^)	Pore Volume (cm^3^ g^−1^)
MSNs	1048	1.51
Polyphosphate-MSNs	10.02	0.0166

**Table 2:** Effect of time and temperature on U adsorption (polyphosphate-modified MSNs).

A. Effect of time on U adsorption at 25 °C.				
t (minute)	Ce (mg L^−1^)	SD	%	qe (mg g^−1^)
5	75.15	1.6	24.85	19.9
10	64.6	0.98	35.4	19.9
15	64.95	0.57	35.05	19.5
30	69.71	2.3	29.59	20.4
60	68.45	1.3	30.86	20.1
120	50.37	0.3	49.12	20.1
180	55.12	1.41	44.32	20.4
240	61.6	0.94	37.78	20.3
B. Effect of temperature on U adsorption at 25 °C.				
UO_2_^2+^ concentration (mg L^−1^)	Ce (mg L^−1^)	SD	%	qe (mg g^−1^)
10	2.54	0.097	73.81	20.5
40	25.37	1.071	33.24	20.3
60	41	0.79	26.79	20
80	53.58	0.26	27.59	19.8
100	68	1.42	31.31	19.7
C. Effect of temperature on U adsorption at 35 °C.				
UO_2_^2+^ concentration (mg L^−1^)	Ce (mg L^−1^)	SD	%	qe (mg g^−1^)
10	4.89	0.081	49.59	20.5
40	25.6	1.43	32.63	19.6
60	41.96	1.87	25.07	20.1
80	20	1.2	72.97	19.5
100	25.39	1.9	74.35	19.7
D. Effect of temperature on U adsorption at 45 °C.				
UO_2_^2+^ concentration (mg L^−1^)	Ce (mg L^−1^)	SD	%	qe (mg g^−1^)
10	1.21	0.086	87.53	19.8
40	22.16	0.86	41.68	19.5
60	33.88	1.43	39.5	20.2
80	53.46	2.72	27.76	19.7
100	62.42	1.32	36.95	19.6
E. Effect of temperature on U adsorption at 55 °C.				
UO_2_^2+^ concentration (mg L^−1^)	Ce (mg L^−1^)	SD	%	qe (mg g^−1^)
10	1.29	0.02	86.7	19.6
40	22.57	0.62	40.61	20
60	36	0.84	35.71	20.2
80	19.17	0.43	74.09	19.7
100	52.5	0.78	46.97	19.8

**Table 3:** Effect of pH on adsorption at two U concentrations (50 and 100 mg L^−1^) at 25 °C.

A. 60 min at U concentration 100 mg L^−1^.					
pH	Ci (mg L^−1^)	Ce (mg L^−1^)	SD	%	qe (mg g^−1^)
2	100	94.64	0.51	5.36	19.8
4	100	76.28	1.2	23.72	19.6
6	100	68.45	1.56	31.55	20
8	100	82.86	0.29	17.14	19.8
B. 60 min at U concentration 50 mg L^−1^.					
pH	Ci (mg L^−1^)	Ce (mg L^−1^)	SD	%	qe (mg g^−1^)
2	50	48	1.02	4	19.5
4	50	31.95	0.33	36.1	19.7
6	50	37.58	1.44	24.84	20.4
8	50	40.55	0.95	18.9	20.1

**Table 4:** Effect of adsorbent dosage on U removal by polyphosphate-functionalized MSNs at room temperature and 60 min contact time.

A. 60 min at U concentration 100 mg L^−1^.				
Weight (mg)	Ce (mg L^−1^)	SD	%	qe (mg g^−1^)
10	63.50	1.05	36.5	9.6
15	66.88	1.51	33.12	14.8
20	68.45	0.86	31.55	20.1
25	64.45	0.3	35.36	24.7
30	61.56	1.45	38.44	29.7
50	65.57	0.67	34.43	49.9
B. 60 min at U concentration 50 mg L^−1^.				
Weight (mg)	Ce (mg L^−1^)	SD	%	qe (mg g^−1^)
10	9.6	0.53	36.34	9.8
15	14.8	1.1	35	15.1
20	20.1	1.4	35.46	20.1
25	24.7	0.96	38.54	24.6
30	29.7	0.43	39.74	30.3
50	49.9	2.33	21.54	50.1

**Table 5:** Selective adsorption of U in multi-metal systems by polyphosphate-functionalized MSNs at room temperature and 60 min contact time.

A. The effect of adsorption with Pb.				
Element	Ce (mg L^−1^)	SD	%	qe (mg g^−1^)
Cr	82	0.49895	18	50
Ni	84	1.05175	16	
Cu	91	1.45105	9	
Zn	89	1.0933	11	
Cd	90	1.17	10	
Pb	84	0.6324	16	
U	60	0.6203	40	
B. The effect of adsorption in the absence of Pb.				
Element	Ce (mg L^−1^)	SD	%	qe (mg g^−1^)
Cr	84	1.333	16	49.7
Ni	81	0.82145	19	
Cu	90	0.8328	10	
Zn	88	1.0487	12	
Cd	95	1.18225	5	
U	63	0.8927	37	

**Table 6:** Langmuir and Freundlich isotherm parameters for U(VI) adsorption onto polyphosphate-functionalized MSNs at room temperature.

Isotherm Model	Parameter	Value
Langmuir	R^2^	0.9697
	$q_{max}$ (mg g^−1^)	114.94
	*K_L_* (L mg^−1^)	0.29
	*R_L_* range	0.04–0.13
Freundlich	R^2^	0.8544
	*K_F_* (mg g^−1^)	57.0426
	*n*	6.2460

## Data Availability

Data and samples of the material are available from the authors.

## References

[1] Bhalara P., Punetha D., Balasubramanian K. (2014). A review of potential remediation techniques for uranium (VI) ion retrieval from contaminated aqueous environment. J. Environ. Chem. Eng..

[2] Shen J., Schäfer A. (2014). Removal of fluoride and uranium by nanofiltration and reverse osmosis: A review. Chemosphere.

[3] Khedr M. (2013). Radioactive contamination of groundwater, special aspects and advantages of removal by reverse osmosis and nanofiltration. Desalination.

[4] Jun B., Lee H., Park S., Kim T. (2021). Purification of uranium-contaminated radioactive water by adsorption: A review on adsorbent materials. Sep. Purif. Technol..

[5] Chakrabortty S., Roy M., Pal P. (2012). Removal of fluoride from contaminated groundwater by cross flow nanofiltration: Transport modeling and economic evaluation. Desalination.

[6] Zhou J., Zhou H., Zhang Y., Wu J., Zhang H., Wang G., Li J. (2020). Pseudocapacitive deionization of uranium(VI) with WO_3_/C electrode. Chem. Eng. J..

[7] Reinoso-Maset E., Ly J. (2016). Study of uranium(VI) and radium(II) sorption at trace level on kaolinite using a multisite ion exchange model. J. Environ. Radioact..

[8] Huynh J., Palacio R., Safizadeh F., Lefèvre G., Descostes M., Eloy L., Guignard N., Rousseau J., Royer S., Tertre E. (2017). Adsorption of uranium over NH_2_-functionalized ordered silica in aqueous solutions. ACS Appl. Mater. Interfaces.

[9] Brahim M., Benettayeb A., Haddou B., Amar I., Baigenzhenov O., Lahouel N., Belkacem M., Khodja S., Hosseini-Bandegharaei A. (2026). Advances in adsorbent materials for uranium removal from waters and wastewaters. J. Hazard. Mater. Adv..

[10] Wang F., Li C., Yang W., Dou W., Niu C., Liu Y., Pan Q. (2019). Efficient removal of U(VI) using functionalized hollow mesoporous silica nanospheres. ChemistrySelect.

[11] Giannakoudakis D., Bandosz T., Balkus T. (2021). Enhanced uranium removal from acidic wastewater by phosphonate-functionalized ordered mesoporous silica: Surface chemistry matters the most. J. Hazard. Mater..

[12] Dressler A., Leydier A., Grandjean A. (2022). Effects of Impregnated Amidophosphonate Ligand Concentration on the Uranium Extraction Behavior of Mesoporous Silica. Molecules.

[13] Xu M., Han X., Wang T., Li S., Hua D. (2018). Conjugated microporous polymers bearing phosphonate ligands as an efficient sorbent for potential uranium extraction from high-level liquid wastes. J. Mater. Chem. A.

[14] Zhang W., Dong X., Mu Y., Wang Y., Chen J. (2021). Constructing adjacent phosphine oxide ligands confined in mesoporous Zr-MOFs for uranium capture from acidic medium. J. Mater. Chem. A.

[15] Thapa R., Tanwar K., Bhatnagar M. (2021). Recovery of uranium with bisphosphonate modified mesoporous silicon. Sep. Purif. Technol..

[16] Parida D., Salmeia K., Sadeghpour A., Zhao S., Maurya A., Assaf K., Moreau E., Pauer R., Lehner S., Jovic M. (2021). Template-free synthesis of hybrid silica nanoparticle with functionalized mesostructure for efficient methylene blue removal. Mater. Des..

[17] Barczak M., Dobrowolski R., Borowski P., Giannakoudakis D. (2020). Pyridine-, thiol- and amine-functionalized mesoporous silicas for adsorptive removal of pharmaceuticals. Microporous Mesoporous Mater..

[18] Al Othman Z., Apblett A.W. (2009). Synthesis of mesoporous silica grafted with 3-glycidoxypropyltrimethoxy-silane. Mater. Lett..

[19] Chircov C., Spoială A., Păun C., Crăciun L., Ficai D., Ficai A., Andronescu E., Turculeƫ Ș. (2020). Mesoporous Silica Platforms with Potential Applications in Release and Adsorption of Active Agents. Molecules.

[20] Alswieleh A., Alshahrani M., Alzahrani K., Alghamdi H., Niazy A., Alsilme A., Beagan A., Alsheheri B., Alghamdi A., Almeataq M. (2019). Surface modification of pH-responsive poly(2-(tert-butylamino)ethyl methacrylate) brushes grafted on mesoporous silica nanoparticles. Des. Monomers Polym..

[21] Muzammil E.M., Khan A., Stuparu M.C. (2017). Post-polymerization modification reactions of poly(glycidyl methacrylate)s. RSC Adv..

[22] Verma S., Kim K. (2021). Graphene-based materials for the adsorptive removal of uranium in aqueous solutions. Environ. Int..

[23] Li Y., He H., Liu Z., Lai Z., Wang Y. (2021). A facile method for preparing three-dimensional graphene nanoribbons aerogel for uranium(VI) and thorium(IV) adsorption. J. Radioanal. Nucl. Chem..

[24] Michailidou G., Koumentakou I., Liakos E., Lazaridou M., Lambropoulou D., Bikiaris D., Kyzas G.Z. (2021). Adsorption of Uranium, Mercury, and Rare Earth Elements from Aqueous Solutions onto Magnetic Chitosan Adsorbents: A Review. Polymers.

[25] da Silva Alves D., Healy B., Pinto L., Cadaval T., Breslin C. (2021). Recent Developments in Chitosan-Based Adsorbents for the Removal of Pollutants from Aqueous Environments. Molecules.

[26] Elwakeel K., Hamza M., Guibal E. (2021). Effect of agitation mode (mechanical, ultrasound and microwave) on uranium sorption using amine- and dithizone-functionalized magnetic chitosan hybrid materials. Chem. Eng. J..

[27] Tural B., Ertaş E., Tural S. (2016). Removal of phenolic pollutants from aqueous solutions by a simple magnetic separation. Desalin. Water Treat..

[28] Alam M., Gorman-Lewis D., Chen N., Safari S., Baek K., Konhauser K., Alessi D. (2018). Mechanisms of the removal of U(VI) from aqueous solution using biochar. Environ. Sci. Technol..

[29] Liang L., Xi F., Tan W., Meng X., Hu B., Wang X. (2021). Review of organic and inorganic pollutants removal by biochar and biochar-based composites. Biochar.

[30] Liu X., Pang H., Liu X., Li Q., Zhang N., Mao L., Qiu M., Hu B., Yang H., Wang X. (2021). Orderly porous covalent organic frameworks-based materials: Superior adsorbents for pollutants removal from aqueous solutions. Innovation.

[31] Yang T., Tian C., Yan X., Xiao R., Lin Z. (2021). Rational construction of covalent organic frameworks with multi-site functional groups for highly efficient removal of low-concentration U(VI) from water. Environ. Sci. Nano.

[32] Nezhad M., Semnani A., Tavakkoli N., Shirani M. (2021). Selective and highly efficient removal of uranium from radioactive effluents by activated carbon functionalized with 2-aminobenzoic acid as a new sorbent. J. Environ. Manag..

[33] Guo H., Mei P., Xiao J., Huang X., Ishag A., Sun Y. (2021). Carbon materials for extraction of uranium from seawater. Chemosphere.

[34] Cheng Y., He P., Dong F., Nie X., Ding C., Wang S., Zhang Y., Liu H., Zhou S. (2019). Polyamine and amidoxime groups modified bifunctional polyacrylonitrile-based ion exchange fibers for highly efficient extraction of U(VI) from real uranium mine water. Chem. Eng. J..

[35] Batuk D., Kalmykov S., Denecke M. (2011). Sorption and speciation of uranium on silica colloids. Actinide Nanoparticle Research.

[36] Alshammari A.S., Almeataq M.S., Basfar A.A. (2023). Treatment of Uranium-Contaminated Ground Water Using Adsorption Technology via Novel Mesoporous Silica Nanoparticles. Molecules.

[37] Sarafraz H., Alahyarizadeh G., Minuchehr A., Modaberi H., Naserbegi A. (2019). Economic and efficient phosphonic functional groups mesoporous silica for uranium selective adsorption from aqueous solutions. Sci. Rep..

[38] Wang Y.L., Zhu L., Guo B.L., Chen S.W., Wu W.S. (2014). Mesoporous silica SBA-15 functionalized with phosphonate derivatives for uranium uptake. New J. Chem..

[39] Al-Anber M.A., Al-Momani I.F., Hijazi A.K., Sagadevan S., Al-Adaileh N.A., Daoud H., Allaham M.M., Sobola D. (2025). Phosphate-doped silica gel nanocomposites for effective uranium ion remediation from water. J. Radioanal. Nucl. Chem..

[40] Foo K.Y., Hameed B.H. (2010). Insights into the modeling of adsorption isotherm systems. Chem. Eng. J..

[41] Zhuang S., Wang J. (2019). Removal of U (VI) from aqueous solution using phosphate functionalized bacterial cellulose as efficient adsorbent. Radiochim. Acta..

[42] Yuan L.Y., Liu Y.L., Shi W.Q., Lv Y.L., Lan J.H., Zhao Y.L., Chai Z.F. (2011). High performance of phosphonate-functionalized mesoporous silica for U(VI) sorption from aqueous solution. Dalton Trans..

